# Pharmacomicrobiomics and type 2 diabetes mellitus: A novel perspective towards possible treatment

**DOI:** 10.3389/fendo.2023.1149256

**Published:** 2023-03-23

**Authors:** Liyang Jia, Shiqiong Huang, Boyu Sun, Yongguang Shang, Chunsheng Zhu

**Affiliations:** ^1^ Department of Traditional Chinese Medicine, The First Affiliated Hospital of Zhengzhou University, Zhengzhou, China; ^2^ Department of Pharmacy, The First Hospital of Changsha, Changsha, China; ^3^ Department of Pharmacy, The Third People’s Hospital of Qingdao, Qingdao, China; ^4^ Department of Pharmacy, China-Japan Friendship Hospital, Beijing, China

**Keywords:** type 2 diabetes mellitus, pharmacomicrobiomics, gut microbiome, antidiabetic drugs, treatments

## Abstract

Type 2 diabetes mellitus (T2DM), a major driver of mortality worldwide, is more likely to develop other cardiometabolic risk factors, ultimately leading to diabetes-related mortality. Although a set of measures including lifestyle intervention and antidiabetic drugs have been proposed to manage T2DM, problems associated with potential side-effects and drug resistance are still unresolved. Pharmacomicrobiomics is an emerging field that investigates the interactions between the gut microbiome and drug response variability or drug toxicity. In recent years, increasing evidence supports that the gut microbiome, as the second genome, can serve as an attractive target for improving drug efficacy and safety by manipulating its composition. In this review, we outline the different composition of gut microbiome in T2DM and highlight how these microbiomes actually play a vital role in its development. Furthermore, we also investigate current state-of-the-art knowledge on pharmacomicrobiomics and microbiome’s role in modulating the response to antidiabetic drugs, as well as provide innovative potential personalized treatments, including approaches for predicting response to treatment and for modulating the microbiome to improve drug efficacy or reduce drug toxicity.

## 1 Introduction

Type 2 diabetes mellitus (T2DM), a major cause of morbidity globally, is a complex disease with environmental and genetic risk factors that ultimately can lead to serious complications (1). It is characterized by peripheral insulin resistance (IR) and impaired insulin secretion (2), and is projected to affect up to 783 million people by 2045 (3). Individuals with T2DM have an increased risk of developing diabetic complications including microvascular events, kidney failure, stroke and limb amputations (4). Although there are several non-pharmacological and pharmacological treatments available for managing T2DM (5, 6), problems associated with potential side-effects and drug resistance remain unresolved.

Over recent years, the human gut microbiota harboring trillions of microbes and other microorganisms forms a complex ecosystem and plays a vital role in health and disease. For instance, gut microbiota functioned as an important contributor in the pathogenesis of obesity and obesity-related metabolic dysfunctions (7). The balance of pathogenic and beneficial bacteria was also reported to be associated with diabetes and cardiovascular diseases (8, 9). Various studies showed the effect of drug intake and drug-induced metabolites on the gut microbiota (10–12), and the gut microbiota could also contribute to an individual’s response to several drugs in turn (13, 14).

Pharmacomicrobiomics, a new branch, has been proposed to describe the influence of microbiome variations on drug response (15). It was useful for investigating how the effect of drugs could be modulated by the gut microbiota. In addition, pharmacomicrobiomics played a crucial role in the development of personalized medicine in order to improve the drug efficacy and reduce adverse drug reactions (16). Undoubtedly, the microbiota modulation associated with pharmacomicrobiomics has the potential to enable the development of microbiota-targeting approaches.

In the present review, we summarize microbiome variations in T2DM and highlight how these microbiomes actually play a preponderant role in its development. Besides, we also investigate pharmacomicrobiomics and microbiome’s role in modulating the response to antidiabetic drugs, focusing particular attention on innovative potential personalized treatments for T2DM.

## 2 The role of gut microbiota in T2DM

Gut microbiota, known as the “human second genome”, consists of the 10–100 trillion microorganisms including bacteria, archaea and viruses (17), and has 150 times larger gene sets than humans (18). It was a well-known fact that the gut microbiota played an crucial role in the proper functioning of human organisms (19). Due to the advancements in sequencing technologies, researches on gut microbiome have developed rapidly during the past decade. Accumulating evidence indicated that gut microbiota dysbiosis contributed to the onset and development of T2DM (20–22).

Although the complete bacterial counts were similar between healthy controls and T2DM patients (23), the diversity was significantly declined in T2DM (10, 24–27). Furthermore, the Integrative Human Microbiome Project found that prediabetic individuals had distinguishable microbial patterns at baseline from the healthy controls (28). Both humans and animal models with T2DM showed the compositional changes in microbiota profiles, especially at the phyla and genus levels (29, 30). A previous study showed a decrease in the abundance of butyrate-producing bacteria and an increase in several opportunistic pathogens, including *Clostridium symbiosum*, *Clostridium hathewayi* and *Escherichia coli* in Chinese T2DM patients (29). Likewise, Li et al. revealed a notable decrease of butyrate-producing bacteria such as *Bifidobacterium* and *Akkermansia*, as well as a significant increase of *Dorea* in Chinese T2DM individuals (31). Another study in Europe found an increase abundance of four *Lactobacillus* species and a reduction in the abundance of five *Clostridium* species in T2DM patients (23). Analogously, a recent study demonstrated that *Lactobacillus* was significantly higher, whereas *Clostridium coccoides* and *Clostridium leptum* were significantly lower in newly diagnosed T2DM patients (32). Furthermore, patients with refractory T2DM revealed reductions in *Akkermansia muciniphila* and *Fusobacterium*, as well as a corresponding enrichment of *Bacteroides vulgatus* and *Veillonella denticariosi* (33). Yassour and his colleagues suggested that decreased *Akkermansia muciniphila* could be used as a biomarker for the early diagnosis of T2DM (34). Notably, *Bacteroidetes*, *Firmicutes* and *Proteobacteria* were reported as the main predominant phyla in T2DM patients (27, 35–37). In newly diagnosed T2DM, the phylum *Firmicutes* significantly increased, along with the phylum *Bacteroidetes* significantly decreased (27, 35, 37). Sedighi et al. performed a case-control study and found that *Firmicutes* increased but *Bacteroidetes* decreased in T2DM patients (24). Uniformly, a recent study recruited 65 T2DM patients and 35 healthy controls and observed a consistent result (36). Interestingly, these studies also highlighted a significant increase (36, 37) or decrease (27, 35) in *Proteobacteria* respectively. Therefore, it is necessary to reduce the impact of confounding factors (i.e. dietary habits, lifestyle, disease status) and increase the sample size to further verify these inconsistent results.

In addition, the role of gut microbiota in T2DM was also confirmed in several animal models (38–40). 16S rRNA gene sequencing illuminated that the abundance of several butyrate-producing bacterial genera, such as *Dialister*, *Anaerotruncus* and some members of *Ruminococcaceae*, was reduced in diabetic cats (38). Okazaki et al. established a T2DM zebrafish model and revealed a lower bacterial diversity than the control (39), which indicated functional similarities in T2DM individuals. Wang et al. constructed Zucker diabetic fatty (ZDF) rats that were fed with Purina Lab Diet to induce obesity-related T2DM and found twelve potential biomarkers of microbial flora and 357 differential metabolites in ZDF rats, among which three flora, *Phocea*, *Pseudoflavonifractor* and *Lactobacillus*, contributed to the perturbation of metabolites (40). Besides, microbiome analysis demonstrated that the time-dependent alterations in the fecal microbiome were associated with age and disease progression of T2DM in ZDF rats (41). Of interest, *Bifidobacterium*, *Lactobacillus*, *Ruminococcus*, and *Allobaculum* were the most abundant genera in 15-week-old rats (41). Leptin receptor-deficient db/db mice were commonly used as T2DM murine models (42). Yu et al. found a significant increase in *Verrucomicrobia* and a significant decrease in *Bacteroidaceae* in T2DM murine model (43). They also showed that the fecal microbiota transplantation (FMT) from T2DM murine transplanted into pseudo-germ-free mice induced an increase in body weight and fasting blood glucose. Another study exhibited a loss of diurnal oscillations in several certain bacteria, including *Akkermansia*, *Bifidobacterium*, *Allobaculum*, and *Oscillospira* in T2DM db/db mice (44). In high-fat diet (HFD)/streptozotocin (STZ)-induced T2DM mice model, genistein could alleviate inflammation and IR by increasing the abundance of *Bacteroides* and *Prevotella* and decreasing the levels of *Helicobacter* and *Ruminococcus*, indicating that the gut microbiota might be a potential target for the treatment of T2DM (45). Recently, increasing evidence showed that several bacterial taxa, including *Akkermansia muciniphila* (46) and *Bacteroides* (47), had consistent trends in T2DM patients and animal models. Collectively, gut microbiota is closely related to the onset and development of T2DM (**Table 1**), as well as may be an important participant in the pathogenesis of T2DM.

**Table 1:** The changes of gut microbiota in T2DM.

Subjects	Methods	Changes in gut microbiota	References
European women with T2DM	Shotgun sequencing	Increased abundance of four *Lactobacillus* species and reduced the abundance of five *Clostridium* species	(23)
Iranian T2DM patients	16S rRNA sequencing	Increased *Firmicutes* and decreased *Bacteroidetes*	(24)
Chinese T2DM patients	Deep shotgun sequencing	A decrease in the abundance of butyrate-producing bacteria and an increase in *Clostridium symbiosum*, *Clostridium hathewayi* and *Escherichia coli*	(29)
Danish T2DM patients	16S rRNA pyrosequencing	Reduced the proportions of *Firmicutes* and *Clostridia*	(30)
T2DM patients from Northern China	16S rRNA pyrosequencing	Decreased butyrate-producing bacteria such as *Bifidobacterium* and *Akkermansia*, as well as increased *Dorea*	(31)
Newly diagnosed T2DM patients from Taiwan	16S rRNA sequencing	A higer level of *Lactobacillus* and a lower level of *Clostridium coccoides* and *Clostridium leptum*	(32)
Patients with refractory T2DM from Taiwan	16S rRNA sequencing	Decreased *Akkermansia muciniphila* and *Fusobacterium*, as well as enriched *Bacteroides vulgatus* and *Veillonella denticariosi*	(33)
Patients with sub-clinical state of T2DM from Korea	Shotgun metagenomes	Decreased *Akkermansia muciniphila*	(34)
Chinese T2DM patients	16S rRNA sequencing	Increased the abundance of Proteobacteria and the ratio of Firmicutes/Bacteroidetes	(36)
Newly diagnosed T2DM patients from India	16S rRNA sequencing	Decreased *Akkermansia, Blautia*,and *Ruminococcus* and increased *Lactobacillus*	(37)
Lean individuals with newly diagnosed T2DM	Shotgun metagenomic sequencing	Decreased the abundance of Akkermansia muciniphila	(46)

T2DM, type 2 diabetes mellitus; ZDF, zucker diabetic fatty; HFD, high-fat diet.

**Figure 1:**
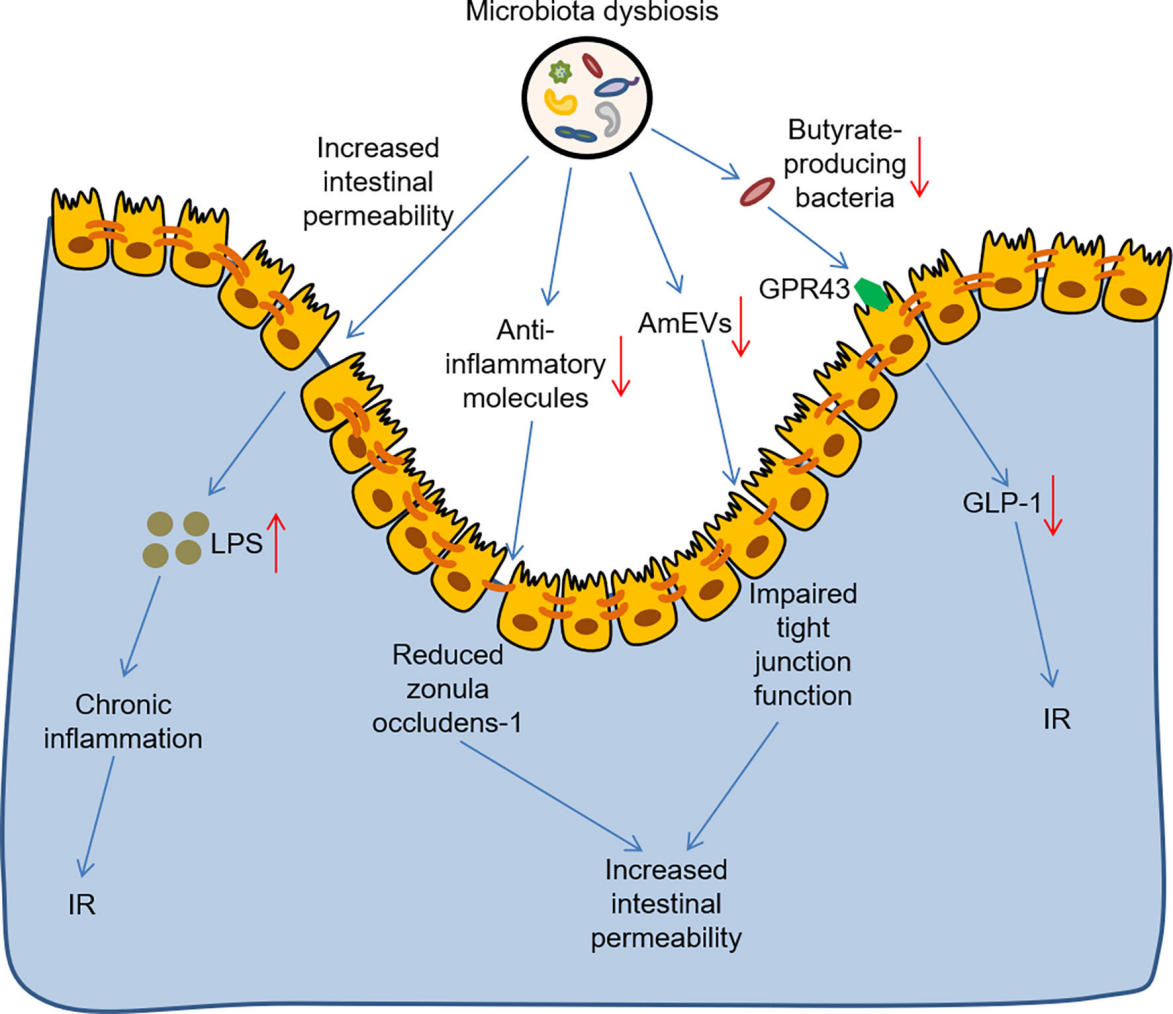
The role of gut microbiota dysbiosis in the development of T2DM. Microbiota dysbiosis increased intestinal barrier permeability and increased the level of LPS, thus leading to chronic inflammation and IR. Microbiota dysbiosis reduced the anti-inflammatory molecule and AmEVs levels, thus impairing the intestinal barrier structure and increasing intestinal barrier permeability. Microbiota dysbiosis also reduced butyrate-producing bacteria, which contributed to IR by inhibiting GLP-1 secretion from colonic L cells *via* the fatty acid receptor GPR43. LPS, lipopolysaccharide; IR, insulin resistance; AmEVs, *Akkermansia muciniphila*-derived extracellular vesicles GLP-1, glucagon-like peptide-1 receptor.

As mentioned above, gut microbiota plays a regulatory role in the development of T2DM. There is growing evidence that microbiota and its metabolites are involved in modulating gut permeability, as well as influence immune and inflammatory responses and metabolic homeostasis in T2DM (**Figure 1**). Intestinal barrier protects the body from intestinal lipopolysaccharide (LPS), and increased intestinal permeability leads to chronic inflammation and is a characteristic of human T2DM (48). A previous study, in turn, verified that hyperglycemia drived intestinal barrier permeability through altering the integrity of tight and adherence junctions (49). Microbial anti-inflammatory molecule derived from *Faecalibacterium prausnitzii* could restore the intestinal barrier structure and function *via* stabilizing the cell permeability and increasing zonula occludens-1 expression in T2DM mouse model (50). *Akkermansia muciniphila*-derived extracellular vesicles (AmEVs) were reported to decrease in the fecal samples of patients with T2DM, and AmEV administration reduced intestinal permeability by enhancing tight junction function and thus improved glucose homeostasis in HFD-induced diabetic mice (51). Strikingly, numerous clinical and preclinical researches have shown that gut microbial imbalance is closely interconnected to IR. For example, an observational study found that reduced fecal *Akkermansia muciniphila* abundance increased the severity of IR in Asians with T2DM, particularly those who were lean in weight (52). Similarly, another study reported that butyrate-producing bacteria, such as *Fecalibacterium prausnitzii*, alleviated IR by inducing glucagon-like peptide-1 receptor (GLP-1) secretion from colonic L cells *via* fatty acid receptor GPR43 (53). Of note, the levels of fecal and serum LPS were elevated in HFD/STZ-induced T2DM model (54). Subsequent studies have confirmed that when LPS is transported to metabolic tissues, it induces a pro-inflammatory response through the activation of toll-like receptor 4 (TLR4) pathway, ultimately leading to IR (55). Moreover, Amuc_1100, a purified membrane protein from *Akkermansia muciniphila*, improved the integrity of the intestinal barrier by interacting with toll-like receptor 2 (TLR2), thus alleviating IR in HFD-fed mice (56). It was well known that microbiota and its metabolites stimulated anti-inflammatory cytokines and decreased inflammatory markers, as well as improved glucose metabolism. For instance, *Lactobacillus plantarum* had potential hypoglycaemic ability and improved glucose metabolism by increasing the levels of interleukin-10 and reducing the levels of malondialdehyde and tumour necrosis factor-α, thus ameliorating IR and systemic inflammation in HFD/STZ-induced T2DM mice (57, 58). Furthermore, *Lactobacillus casei* and *rhamnosus* also decreased the levels of the inflammatory markers tumor necrosis factor-α and interleukin-6 in HFD/STZ-induced T2DM rats (59, 60), thereby improving glucose metabolism and attenuating symptoms of T2DM. Although several potential detrimental microbes, such as *Fusobacterium nucleatum* and *Ruminococcus gnavus* could increase several inflammatory cytokines in inflammatory diseases (61, 62), its similar role in T2DM remained to be further investigated. Taken together, more studies are needed to deepen our understanding of the role of gut microbiota in T2DM.

## 3 Pharmacomicrobiomics focuses on T2DM

**Figure 2:**
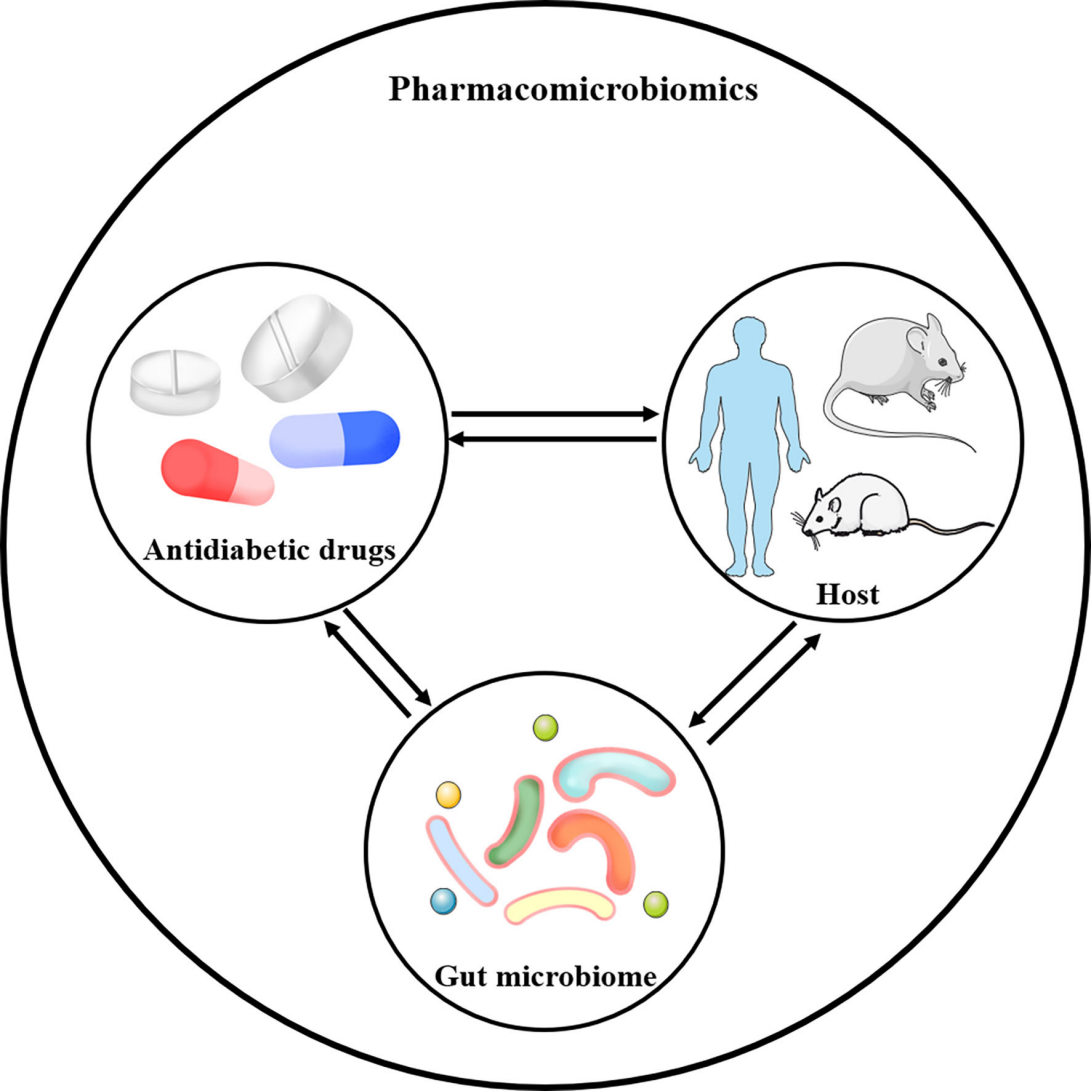
Pharmacomicrobiomics studies drug-microbe-host interactions. Antidiabetic drugs-microbe interactions could result in alterations in microbial composition and changes in the chemical structure of compounds, which could in turn directly or indirectly affect the drug response in host, including T2DM patients, mice and rats.

Given that the preponderant role of gut microbiota in T2DM, there is growing interest in pharmacomicrobiomics and microbiome’s role in T2DM. Pharmacomicrobiomic studies have been proposed to describe the bidirectional effects between the gut microbiome and antidiabetic drugs, including metformin, thiazolidinedione (TZD), α-glucosidase inhibitors (α-GIs), sodium-glucose cotransporter 2 (SGLT2) inhibitors, glucagon-like peptide-1 receptor agonists (GLP-1 RAs), dipeptidyl peptidase-4 (DPP-4) inhibitors and traditional Chinese medicines (TCMs), appropriately investigating the interactions between the host, gut microbiome and drug action (**Figure 2**).

### 3.1 Metformin-microbiome-host interactions

Metformin, the most commonly used glucose-lowering drug, can alleviate patients’ hyperglycemia *via* the suppression of hepatic glucose production and the increase of glucose uptake and utilization in adipocytes and muscle cells (63). A vast body of studies revealed that metformin altered the gut microbiota community in T2DM (11, 64–66). A multicenter, randomized clinical trial suggested that metformin ameliorated hyperglycemia and hyperlipidemia in T2DM patients *via* increasing beneficial bacteria, such as *Blautia* and *Faecalibacterium* (64). Another randomized, placebo-controlled study showed that metformin perturbed the gut microbiome in individuals with treatment-naive T2DM (11). The authors also transplanted the fecal samples from donors (treated with metformin for 4 months) to germ-free mice and observed that glucose tolerance was improved by increasing the production of short-chain fatty acids (SCFAs) or altering plasma bile acid composition, suggesting a direct metabolic benefits of metformin. Similarily, Sun et al. demonstrated that *Bacteroides fragilis* was decreased and the bile acid glycoursodeoxycholic acid (GUDCA) was increased in newly diagnosed T2DM individuals treated with metformin, and the benefits of metformin were abrogated in HFD-fed mice colonizaed with *Bacteroides fragilis*, implicating that *Bacteroides fragilis*–GUDCA–intestinal farnesoid X receptor (FXR) axis mediated the glucose-lowering effect of metformin (67). A recent systematic review disclosed that pre-diabetes and newly diagnosed T2DM patients treated with metformin were correlated with increases in specific taxa associated with metabolic control, such as *Enterobacteriales* and *Akkermansia muciniphila* (68). In line with clinical research, studies in animal models further confirmed that metformin increased SCFAs production, reduced circulation lipopolysaccharides and inhibited intestinal proinflammatory signaling activities (65, 69, 70), thus contributing to improving metabolic disoders. Notably, gut microbiota could also mediate the side effects of metformin. Forslund et al. emphasized that a relative increase in abundance of *Escherichia* could enrich virulence factors and gas metabolism genes (10), which contributed to the gastrointestinal side effects of metformin.

### 3.2 TZD-microbiome-host interactions

TZD drugs belong to peroxisome proliferative activated receptor (PPARG) agonists and improve insulin sensitivity for T2DM (71). It reduced hepatic glucose production and increased peripheraltion of glucose and lipid metabolism, thus improving glycemic control. Few studies have discussed the interaction between gut microbiome homeostasis and insulin sensitizers and insulin in T2DM (72, 73). Full-length bacterial 16S rRNA sequencing and RNA sequencing analysis presented that rosiglitazone improved insulin sensitivity without altering the composition of gut microbiome but modifying gene expression signatures associated with lipid and carbohydrate metabolism as well as immune regulation in diabetic mice (72). Moreover, insulin improved taurine and hypotaurine metabolism *via* increasing *Fusobacterium* and up-regulating the genes involved in triglyceride and arachidonic acid metabolism (73).

### 3.3 α-GIs-microbiome-host interactions

α-GIs, including acarbose, voglibose and miglitol, are considered to postpone the digestion of carbohydrates in the intestinal tract and reduce postprandial hyperglycemia in noninsulin-dependent T2DM (74). They are commonly used oral glucose-lowering drugs in China and many Asian countries. A randomized clinical study revealed that acarbose increased the abundance of *Bifidobacterium*, *Eubacterium* and *Lactobacillus*, and lowered the abundance of *Bacteroides* in Japanese patients with T2DM (75). Likewise, in Chinese patients with T2DM, Su et al. found that acarbose treatment increased the content of *Bifidobacterium* and *Enterococcus*, as well as decreased some inflammatory cytokines (76). Mechanistically, Gu et al. highlighted that acarbose altered the relative abundance of microbial genes involved in bile acid metabolism and improved metabolic parameters (12). Interestingly, acarbose also increased the relative abundance of *Ruminococcus* and *Bifidobacterium* in ZDF rats (77). On the other hand, acarbose was an inhibitor of both human and bacterial α-glucosidases, which might limite the ability of the target microbiome to metabolize complex carbohydrates, thus leading to the resistance of acarbose (78). Additionally, due to the weakened microbial enzyme activities, the metabolism of voglibose was reduced, along with significantly glucose-lowering effects were presented in antibiotic pretreatment mice (79), suggesting that gut microbiota mediated the effect of α-glucosidase inhibitors.

### 3.4 SGLT2 inhibitors-microbiome-host interactions

SGLT2 is expressed in the renal proximal tubule and accounts for reabsorbing the filtered glucose. SGLT2 inhibitors exert the glucose-lowering effect by blocking glucose reabsorption in the renal proximal tubule and increasing urinary glucose excretion, accompanied with pleiotropic benefits in cardiovascular and renal protection (80, 81). Several studies have explored the alteration of gut microbiota with SGLT2 inhibitor treatment (82–85). After a 3-month intervention, empagliflozin improved cardiovascular disease (CVD) risk factors in patients with T2DM, which might be attributed to the significantly altered gut microbiota, including the elevated levels of SCFA-producing bacteria (*Roseburia*, *Eubacterium*, and *Faecalibacterium*) and a reduction in several harmful bacteria (*Escherichia*–*Shigella*, *Bilophila*, and *Hungatella*) (82). Whereas, van Bommel et al. reported that 2-week treatment with dapagliflozin and gliclazide did not affect either gut microbiome alpha diversity or composition in T2DM patients treated with metformin (83). This discrepancy might be due to the fact that all the participants had already been treated with metformin, which could overshadow the possible impact of dapagliflozin on the gut microbiome. In T2DM mice, dapagliflozin treatment showed a trend for increased *Akkermansia muciniphila* and decreased *Oscillospira* and *Firmicutes*/*Bacteroidetes* ratios (84). However, another study demonstrated that dapagliflozin did not increase the abundance of beneficial bacteria (85). Therefore, more rigorous clincial studies with greater sample size are needed to figure out the interactions between SGLT2 inhibitors and gut microbiota.

### 3.5 GLP-1 RAs-microbiome-host interactions

GLP-1 secreted by enteroendocrine L cells is an incretin hormone and stimulates glucose-dependent insulin secretion. GLP-1 RAs, a new type of hypoglycemic drugs, mimic the effects of endogenous GLP-1, as well as improve glycemic control and cardiovascular outcomes for T2DM patients (86, 87). Accumulating evidence reported that GLP-1 RAs were linked to the changed composition of gut microbiota (88–91). In liraglutide-treated diabetic male rats, several SCFA-producing bacteria, such as *Bacteroides*, *Lachnospiraceae*, and probiotic bacteria, *Bifidobacterium*, were selectively enhanced, which might alleviate systemic inflammation and improve glucose control (88). Besides, germ-free mice colonized with microbiota from liraglutide-treated diabetic mice were shown to improve glucose-induced insulin secretion and regulate the intestinal immune system (91). Also, in T2DM patients, microbial interaction network was altered in patients treated with liraglutide. The distribution of community structure differed between the pre-liraglutide-treatment group (21 species of bacteria were abundant) and post-liraglutide-treatment group (15 species were abundant) (89). Nevertheless, a recent study enrolling 51 T2DM adults with initial therapy of metformin and/or sulphonylureas showed that the diversity and composition of the intestinal microbiota did not change after 12-week liraglutide intervention (92). This inconsistency might be attributed to the initial therapy of metformin and/or sulphonylureas, which could counteract the effect of liraglutide. Recently, Tsai et al. found that gut microbiota contributed to the heterogenicity of GLP-1 RA responses in T2DM patients (90). To sum up, the positive microbial signatures, mainly including *Bacteroides* and *Roseburia*, with immunomodulation effects were dominant in GLP-1 RA responders, while the negative microbial signatures, such as *Prevotella*, *Butyricimonas*, *Mitsuokella* and *Dialister*, with pro-inflammatory properties were dominant in GLP-1 RA non-responders (90). Thus, gut microbiota may be a potential target to improve the GLP-1 resistance.

### 3.6 DPP-4 inhibitors-microbiome-host interactions

DPP-4 inhibitors improve hyperglycemic conditions by stabilizing GLP-1 and glucose-dependent insulinotropic polypeptides (93). A series of studies considered that DPP-4 inhibitors reshaped the microbial composition and increased fecal SCFAs to improve metabolic homeostasis (94–97). Liao et al. demonstrated that DPP-4 inhibitors promoted a functional shift of the altered microbiome induced by HFD, especially increasing the abundance of *Bacteroidetes*, which contributed to improving glucose homeostasis (94). Another study revealed that DPP-4 inhibitors displayed significantly decreased *Firmicutes*/*Bacteroidetes* ratios, and elevated levels of butyrate-producing *Ruminococcus* and *Dorea* in HFD-induced mice (97). Likewise, vildagliptin treatment also reduced the ratio of *Fimicutes*/*Bacteroidetes*, and increased butyrate-producing bacteria, including *Baceroides* and *Erysipelotrichaeae*, in HFD-induced SD rats (96). Furthermore, vildagliptin significantly reduced DPP-4 activity mainly by decreasing *Oscillibacter* and increasing *Lactobacillus* (95), which provided new therapeutic uses of DPP-4 inhibition to tackle gut microbiome dysfunctions in T2DM.

### 3.7 TCMs-microbiome-host interactions

TCMs, known as botanical medicine or phytomedicine, could significantly improve glucose control by enhancing insulin sensitivity, simulating insulin secretion and protecting β-cells (98). In recent years, increasing evidence confirmed that TCMs improved glucose metabolisms and alleviated T2DM at least partly by modulating gut microbiota (99).

**Figure 3:**
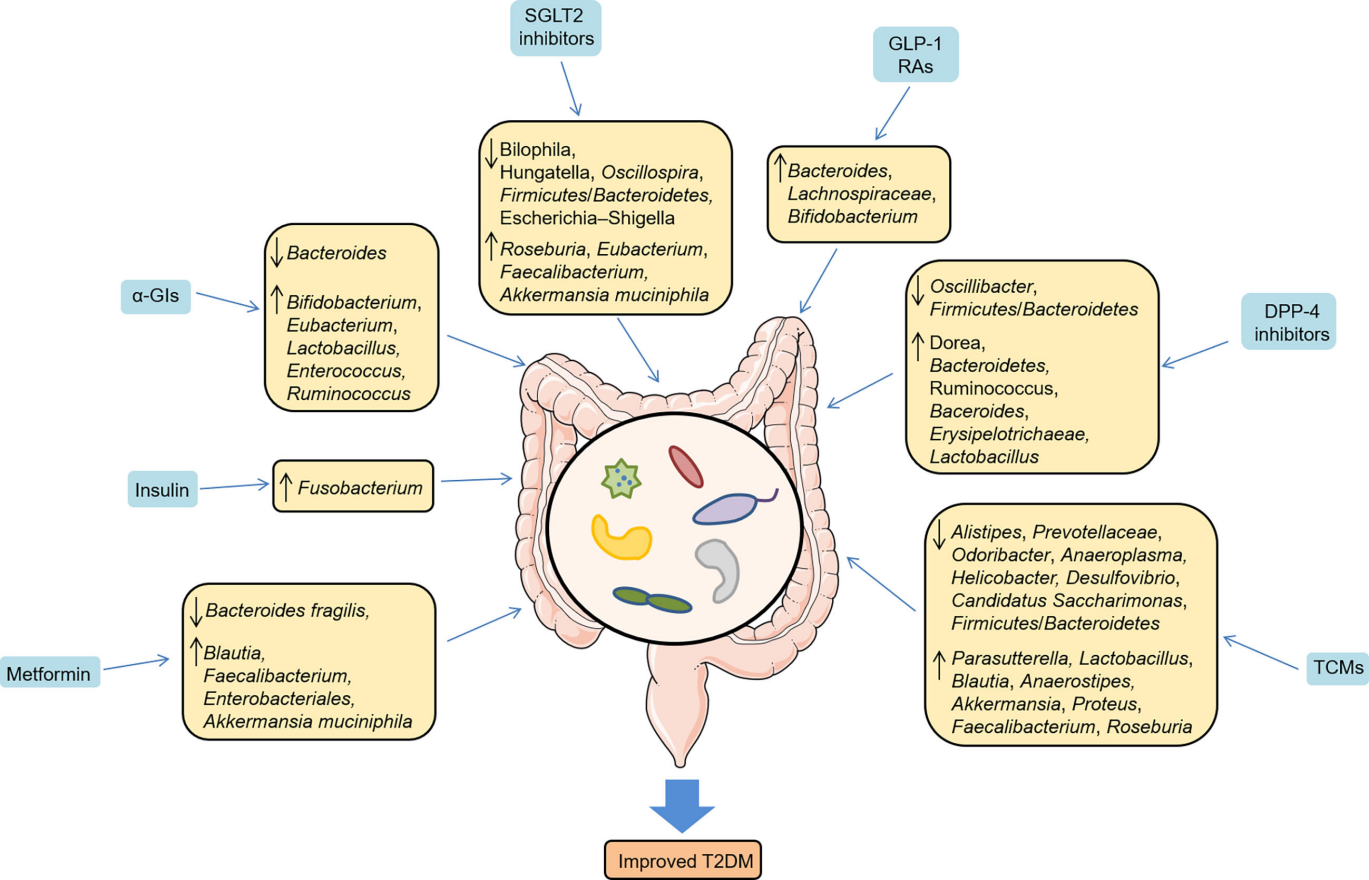
Antidiabetic drugs regulated the relative abundance of gut microbes and improved T2DM.

A number of studies in animal models of T2DM have extensively explored the interactions between TCMs and gut microbiota (100–107). Zhou et al. found that ginsenoside Rb1, one of the most valuable herbal medicine, increased the abundance of *Parasutterella*, and decreased *Alistipes*, *Prevotellaceae*, *Odoribacter* and *Anaeroplasma* in T2DM mice model, thus attenuating IR and metabolic disorders (100). In T2DM rats model, Baihu Rensheng decoction (BHRS) increased the relative abundance of *Lactobacillus*, *Blautia*, and *Anaerostipes*, as well as decreased the *Allobaculum*, *Candidatus Saccharimonas*, and *Ruminococcus* (101). Mechanically, BHRS was considered to repair gut barrier and inhibit TLR4/NF-κB-mediated inflammatory response. Similarly, Buyang Huanwu decoction (BYHWD), a widely used TCM formula, decreased the *Firmicutes*/*Bacteroidetes* ratio and increased the abundance of *Lactobacillus* and *Blautia* (102). Another study suggested that Fufang Fanshiliu decoction enriched the abundance of *Lactobacillus*, *Akkermansia*, and *Proteus*, and reduced the abundance of *Alistipes*, *Desulfovibrio*, and *Helicobacter* in T2DM rats model (104). Moreover, Liu-Wei-Di-Huang Pills improved glucose metabolism by promoting the abundance of *Lactobacillus*, *Allobaculum*, and *Ruminococcus*, and increasing SCFAs levels in T2DM rats model (106), which might be related to the SCFAs-GPR43/41-GLP-1 pathway. Shenqi compound (SQC), a TCM formula, has been widely used for T2DM. It showed that SQC exerted a beneficial role by decreasing the *Firmicutes*/*Bacteroidetes* ratio and modulating metabolites in different pathways (107). Gegen Qinlian Decoction exerted the glucose-lowering effect by significantly modulating the overall gut microbiota structure and enriching butyrate-producing bacteria, including *Faecalibacterium* and *Roseburia*, which subsequently attenuated intestinal inflammation (108). Ge-Gen-Jiao-Tai-Wan formula alleviated symptoms ofT2DM rats by increasing the beneficial phylum *Firmicutes* and bile-acid-related genus *Lactobacillus*, thus promoting the production of primary bile acids, and upregulating the PBA-FXR/TGR5-GLP-1 pathway (109). Intriguingly, a current study suggested that the Scrophulariae Radix and Atractylodes sinensis (XC) pair could assist metformin in improving postprandial hyperglycemia by inhibiting the increase of *Bacteroides* in T2DM rats model (105), which could effectively apply to clinical practice in treating T2DM. In addition to the animal studies, a clinical trial in newly diagnosed T2DM patients also underlined that the hypoglycemic effect of berberine was related to the inhibition of DCA biotransformation by *Ruminococcus bromii* (110). Collectively, these findings address the effect of antidiabetic drugs on gut microbiota (**Figure 3**) and emphasize the host-microbe-drug interactions, providing promising microbiome-targeting approaches to treat T2DM.

## 4 Innovative therapeutics and translational implications of pharmacomicrobiomic studies in T2DM

**Figure 4:**
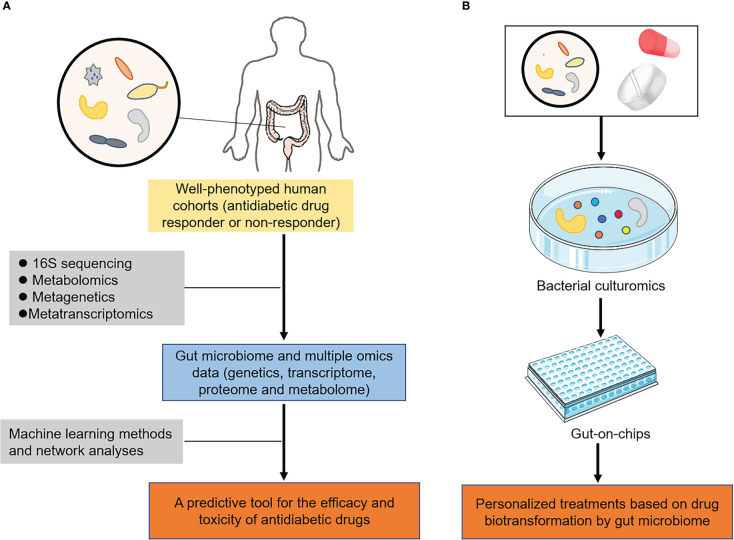
Innovative therapeutics and translational implications of pharmacomicrobiomic studies in T2DM. **(A)** In well-phenotyped patient populations, microbial features and multiple omics could be integrated *via* machine learning and network analyses to predict the efficacy and toxicity of antidiabetic drugs. **(B)** Novel technologies, including organs-on-chips and bacterial culturomics assessed their biotransformation by gut microbiome, providing new insights into personalized medicine in T2DM.

With the host-microbe-drug interactions in mind, the innovative therapeutics and translational applicability of pharmacomicrobiomics are highly relevant to our understanding of drug efficacy and adverse reactions in T2DM (**Figure 4**). Given that the magnitude of response to antidiabetic durgs is known to have a unpredictable and high interindividual variability, personalized treatments based on novel technologies and features of the gut microbiome can help to guide a more rational use of these treatments.

### 4.1 Developing predictive tools *via* machine learning and network analyses

With the completion of the human genome and the human microbiome projects (111), a number of large biobanks including gut microbiome and multiple omics data (information on genetics, transcriptome, proteome and metabolome) had been established, such as UK biobank and TwinsUK cohort (112, 113). These biobanks utilized clinical studies, involving well-characterized human cohorts with extensive clinical and demographic details, exploring the host-microbe-drug interactions. In parallel with the existing data, there was continuous need for digging deeper into the unknown filed of drug-microbiome interactions. The accumulated data in literature calls for the construction of predictive tools or models that consider all such parameters to provide accurate predictions (114, 115). Machine learning methods and network analyses, including decision-tree algorithms and random forest, could then be applied to create a predictive tool for the efficacy and toxicity of antidiabetic drugs in T2DM (**Figure 4A**). For instance, the T2DM prediction model based on the characteristics of the salivary microbiota (microbiome data) was established by random forest in elderly patients with T2DM (116). Another study showed that machine learning tools with gut microbiome profiling exhibited the highest overall predictive power for improving early prediction of T2DM (117). These findings not only had the ability to rapidly inform clinical practice but also elucidated hypotheses regarding the mechanisms in which microbial transformations of drugs changed their pharmacokinetic properties.

### 4.2 Novel technologies for developing personalized treatments

Given the interplay between the host, gut microbiome and drug metabolism, there is increasing awareness that we should take microbiome profile based on novel technologies into account when considering personalized medicine.

Considering that microbiome profiling of human samples provided evidence for microorganism-mediated drug metabolism, further experimental studies are required to identify the specific microbiome responsible for drug biotransformation. Experimental manipulations of gut microbiota incorporated the use of humanized gnotobiotic mice models to further investigate the specific role of the microbiota in modulating drug pharmacokinetics (118). Humanized gnotobiotic mice are typically either germ-free animals or those colonized with defined microbiota and achieved by transplanting human faecal microbiota into germ-free mice (119). As discussed, these models have already proven successful for the treatment of several diseases, including T2DM (120). In recent years, organs-on-chips and bacterial culturomics as emerging technologies also have been developed (121, 122), making functional validation of gut microbiome finally possible. Antidiabetic drugs of interest can be incubated *via* these technologies to assess their biotransformation by gut microbiome, enabling the development of personalized medicine in T2DM (**Figure 4B**).

## 5 Concluding remarks and future perspectives

There is a mountain of evidence linking gut microbiota to T2DM and its hypoglycemic therapy. In recent years, a growing body of research now focuses on the bidirectional effects between the gut microbiome and antidiabetic drugs (123, 124). In this review, we summarize the microbe-drug-host interactions and provide a novel perspective towards possible personalized treatment for T2DM.

With the advancement of the studies on the pharmacomicrobiomics (interactions between drugs, microbial communities and host) (125–127), manipulation of microbiota can be a promising target to improve therapeutic outcomes and alleviate adverse drug effects in T2DM. For instance, prebiotics could modulate intestinal microbiota and increase the relative abundance of beneficial bacteria including SCFAs (128, 129), and the combination of hypoglycemic drugs and certain prebiotics could enhance the glucose-lowering effects (130). In addition, FMT, a process of transferring stool from a healthy donor or antidiabetes treatment subjects to mice, displayed a significant improvement in microbial composition and metabolic homeostasis (131). A prospective study revealed that FMT could bring benefits for the management of T2DM *via* modulating levels of certain microbiome such as *Rikenellaceae* and *Anaerotruncus* (132).

Given the great diversity of microbial signatures and the complex drug-microbe-host interactions, a systems-based approach including the integration of multi-omics data with microbiome data and the utilization of bacterial culturomics are required to understand the underlying mechanisms, thus exploring the new therapeutic interventions and potential personalized strategies.

## Author contributions 

LJ and SH contributed towards the concept and manuscript writing. BS, YS and CZ revised and supervised overall project. All authors read and approved the final version of manuscript. All authors contributed to the article and approved the submitted version.
